# The Role of Secretory Pathways in *Candida albicans* Pathogenesis

**DOI:** 10.3390/jof6010026

**Published:** 2020-02-24

**Authors:** Christiane Rollenhagen, Sahil Mamtani, Dakota Ma, Reva Dixit, Susan Eszterhas, Samuel A. Lee

**Affiliations:** 1 Medicine Service, White River Junction VA Medical Center, White River Junction, VT 05009, USA; Christiane.Rollenhagen@dartmouth.edu (C.R.); sahil.mamtani@gmail.com (S.M.); Dakota.C.Ma.22@dartmouth.edu (D.M.); Reva.Dixit.22@dartmouth.edu (R.D.); Susan.K.Eszterhas@dartmouth.edu (S.E.); 2 Department of Medicine, Geisel School of Medicine at Dartmouth, Hanover, NH 03755, USA; 3 Dartmouth College, Hanover, NH 03755, USA

**Keywords:** *Candida albicans*, biofilm, filamentation, pathogenesis, secretion, trafficking, virulence

## Abstract

*Candida albicans* is a fungus that is a commensal organism and a member of the normal human microbiota. It has the ability to transition into an opportunistic invasive pathogen. Attributes that support pathogenesis include secretion of virulence-associated proteins, hyphal formation, and biofilm formation. These processes are supported by secretion, as defined in the broad context of membrane trafficking. In this review, we examine the role of secretory pathways in *Candida* virulence, with a focus on the model opportunistic fungal pathogen, *Candida albicans*.

## 1. Introduction

The fungus *Candida albicans* is a commensal organism and part of the human microbiome. In certain circumstances, it can become an opportunistic pathogen and has, therefore, been used as a model of fungal pathogenesis [1]. *C. albicans* virulence requires an intact secretory pathway in order to (i) secrete virulence-related proteins, including secreted aspartyl proteases (Saps) and lipases, which are degradative enzymes that assist in tissue invasion, (ii) permit the highly orchestrated process of polarized secretion during hyphal formation, and (iii) support biofilm formation, which promotes intravascular infection and dissemination [2,3,4,5,6,7,8,9].

**Figure 1:**
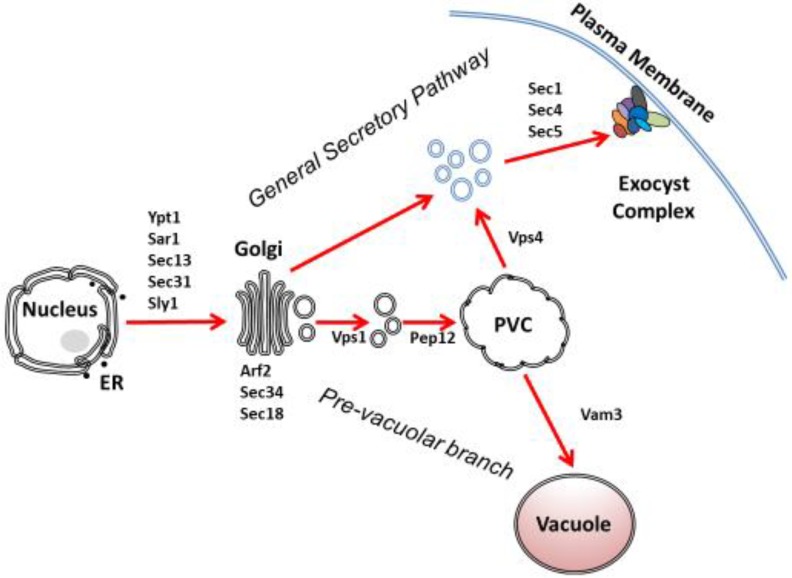
Schematic of antegrade secretory traffic *C. albicans*. In this simplified diagram, two branches of the exocytic pathway are shown: the general secretory pathway and the pre-vacuolar branch of the exocytic pathway. Multiple endocytic and retrograde sorting pathways are not shown in this diagram. In the pre-vacuolar secretory pathway, Vps1p is a dynamin-like GTPase that mediates vesicle budding from the late Golgi. Vps4p is an AAA-type ATPase that mediates vesicle budding from the pre-vacuolar compartment (PVC). Pep12p is a pre-vacuolar t-SNARE (soluble N-ethylmaleimide-sensitive factor activating protein receptor) and Vam3p is a vacuolar t-SNARE. The exocyst complex regulates the critical final steps of polarized secretion just prior to exocytosis, shown in greater detail in Figure 2.

Protein secretion in *C. albicans* requires translocation across membranes of intracellular compartments and the cell wall. Newly synthesized proteins are translocated from ribosomes located in the cytoplasm into the endoplasmic reticulum (ER) via the Sec61p complex which forms a pore structure [10]. The ER is comprised of membranous cisternae and tubules and is connected with the nuclear membrane [11]. In the ER, secreted proteins undergo a variety of processing steps such as folding and glycosylation. The initial transport step from the ER requires the formation of small membrane vesicles that contain the correctly folded protein [12,13]. After a carrier vesicle is formed, fusion is mediated by the vesicle’s soluble N-ethylmaleimide-sensitive factor activating protein receptor (v-SNARE) interacting with the target’s t-SNAREs [14,15,16]. In order for a protein to be secreted, it must then pass through the Golgi complex. The carrier vesicle enters the *cis* region and exits from the *trans* region of the Golgi [17,18]. There, the protein undergoes more glycosylation steps, which protect the exported protein from rapid proteolytic degradation [17]. The post-Golgi secretory vesicle moves to the plasma membrane by transport along microtubules [19]. Exocytic proteins are sorted and transported by a general secretory or pre-vacuolar secretory pathway (Figure 1) [20,21]. When the secretory vesicle reaches the plasma membrane, it fuses with the plasma membrane. This process is called exocytosis, which is mediated by the exocyst and facilitated by the ESCRT complexes [22,23]. The final fusion step results in the release of protein into the extracellular space.

Extracellular vesicles, e.g., the exosome, are capable of transferring signaling molecules and other proteins back into the cell, to the cell membrane, as well as to mammalian host cells. Exosomes are formed and transported as part of the endosomal network and are released upon fusion of multivesicular bodies with the plasma membrane [24,25]. The ER and the trans-Golgi network reinforce the preservation of the endosomal network, which promotes the exchange of membrane components, provide enzymes, and assist with signaling [26]. Transport mechanisms for both exocytosis and endocytosis are complex and use partially overlapping protein complexes that facilitate these processes. This review will primarily focus on the presentation of research findings obtained within the past decade and incorporate these data with the general understanding of the secretory pathway. This subject has been reviewed previously in several excellent reviews focused on the model yeast *Saccharomyces cerevisiae* and *C. albicans* [27,28]. 

### Genomic Overview and Comparison to S. cerevisiae and Other Fungi

*C. albicans* is thought to have over 200 genes encoding for proteins in its complex secretory pathway [28,29,30]. As described in the comprehensive review by Fonzi [28], introns are found in approximately 8% of *C. albicans* secretory apparatus genes, which is similar to the overall frequency of introns in *C. albicans*, implying that there is no selective pressure to keep these introns [28,31]. Furthermore, of the *C. albicans* secretory genes containing introns, only a third of the orthologs in *S. cerevisiae* contain an intron [28,31]. Most components of the secretory pathway are well-conserved in fungi and higher eukaryotes [16,32,33]. However, the presence of homologous genes does not necessarily imply identical or similar function. Moreover, gene regulation can differ significantly among eukaryotic species [27]. 

The Rab proteins provide an interesting example of differences in polarized secretion amongst fungal species. Rab proteins are the largest family of small GTP-binding proteins and play a fundamental role in the fusion of transport vesicles to their targeted membranes. At least eleven *S. cerevisiae* genes encoding Rab proteins have been identified with important roles in vesicle transport [34]. For example, Sec4p is required in vesicle traffic and has functions during the last stage of the secretory pathway in yeast. Without the Sec4p function, cells accumulate Golgi-derived secretory vesicles. This gene is conserved in *C. albicans* and retains a similar function [35,36]. More recently, additional *C. albicans* genes encoding Rab proteins (e.g., *YPT72*, *VPS21*, *BET4*, *YPT53*, *YPT52*) have been identified [37,38,39]. Filamentous fungi other than *C. albicans* have a much larger set of Rab proteins [16,32]. They are thought to recycle endosome membranes to the plasma membrane, contributing to sustained and rapid apical growth of hyphae. These findings suggest differences in polarized secretion and apical growth mechanisms between *C. albicans* other filamentous fungi [16,32,33].

## 2. Translocation and ER Transport

### 2.1. Translocation

Proteins processed through the secretory pathway are synthesized from ribosomes, modified and folded in the endoplasmic reticulum (ER), then packaged in the Golgi apparatus. The ER is a transmembrane network that regulates the production of proteins, ultimately necessary for the maintenance of cell wall integrity, development of hyphae, biofilm formation, and virulence in *C. albicans* [40]. Secreted proteins are transported into the ER for further processing through two observed mechanisms: co-translational translocation and post-translational translocation. 

### 2.2. Co-Translational Translocation

Co-translational translocation involves the direct translation of proteins into the endoplasmic reticulum. An N-terminal hydrophobic signal sequence synthesized in the ribosome targets the emerging polypeptide for the ER and is recognized by a signal recognition particle (SRP), a functionally conserved ribonucleoprotein complex encoded by *SRP101* corresponding to *orf19.3952* in the *Candida* Genome Database [41,42,43,44]. In *S. cerevisiae*, SRP is comprised of Srp9p, Srp14p, Srp21p, Srp19p, Srp54p, Srp68p, Srp72p, and Sec65p subunits [27,45,46,47]. The Srp54p subunit contains a binding site for the N-terminal signal sequence and exists in a GTP-bound form when attached to the nascent polypeptide [47,48]. *SEC65*, a homolog of the central domain of mammalian *SRP19*, is well-conserved and required for the viability of *C. albicans* and *S. cerevisiae*, functioning in SRP assembly [41,45,46,49,50]. 

The SRP-ribosome complex then interacts with the SRP receptor (SR) in a GTP-dependent process at the cytosolic side of the ER membrane. The SR is a heterodimeric, membrane-anchored complex composed of a peripherally associated SRα GTPase and a transmembrane SRβ GTPase [51]. In *S. cerevisiae*, the C-terminus of SRα binds Srp54, bringing the ribosome-polypeptide complex to the ER [47,48,50]. GTP hydrolysis due to the reciprocal GTPase-activating protein (GAP) activities of SRα and Srp54p dissociates the SR-SRP complex, allowing the ribosome to dock at an open translocon on the ER membrane and continue translation into the ER lumen [47]. This process has not been studied explicitly in *C. albicans*, although both the SRP and SR are highly conserved among eukaryotes [50]. 

The translocon is a transmembrane channel that facilitates the translocation and entry of nascent proteins into the ER. The central component of the translocon in *C. albicans* lies in the heterotrimeric Sec61p complex, which includes α, β, and γ subunits [10,51,52,53]. The Sec61p complex is essential for survival, as a mutant with a repressed *MET3* promoter controlling *SEC61* expression exhibited defects in growth and viability [10,27,54]. The specific function of the Sec61p complex in *C. albicans* has otherwise not been directly studied, though the Ca Sec61p complex has a high level (67%) of phylogenetic conservation with the *S. cerevisiae* homolog [10,55]. In *S. cerevisiae*, the Sec61 transmembrane complex is held in a ribosome-free, idle closed conformation but changes into an open state when the ribosome-nascent protein complex docks at the translocon [50].

### 2.3. Post-Translational Translocation

Post-translational translocation involves the transport of a secreted protein into the ER after the protein has been fully translated. Unlike co-translational translocation, post-translational translocation is independent of SRP and is mediated primarily through cytosolic and ER molecular chaperones [41,56,57,58]. The process was initially discovered and extensively studied in *S. cerevisiae*, but specific participants in post-translational translocation have been identified in *C. albicans*, particularly the heat shock protein 70 (Hsp70p) family, a modified Sec61p complex, and Kar2p [10,59].

In *C. albicans*, *HSP70* includes four subfamilies of molecular chaperone genes, *SSA, SSB, SSC,* and *SSD* [60,61]. In *S. cerevisiae*, Ssa proteins are encoded by *SSA1-4* genes and demonstrate over 70% homology with *C. albicans SSA* genes [61,62]. Deletion of *S. cerevisiae (Sc) SSA1* results in a growth defect and cytosolic accumulation of preproteins, specifically preproalpha protein [56,57,63,64]. Both *SSA1* and *SSA2* are expressed on the *C. albicans* cell wall surface and may play some role in virulence [65]. Ssb proteins are encoded by *SSB1* and *SSB2* genes that show over 80% homology with *Sc SSB* genes and act as molecular chaperones for translating ribosomes [60,66]. *C. albicans* cell wall Ssa proteins contribute to virulence by interacting with histatin 5, a fungicidal protein found in human saliva [67]. These proteins of the Hsp70p family function also as extracellular proteins and are involved in biofilm formation, cell nutrient acquisition and cell wall integrity maintenance. In *C. albicans* they are also highly immunogenic, similar to other cell wall proteins such as Bgl2p or Mp65p [68,69,70,71]. These findings support the notion that proteins including Hsp70p found in extracellular vesicles play important roles in host-pathogen communication [65] *C. albicans MSI3* is a homolog of *ScSSE* and functions as a nucleotide exchange factor for Ssa proteins [65,72]. In yeast, post-translationally translocated proteins traverse the ER membrane through the Sec61 complex and an additional Sec62-Sec63 complex, which includes Sec62, Sec63, Sec71, and Sec72 subunits [73,74,75]. The Sec61 translocation pore is present in both co- and post-translational translocation [52,58,76]. While studies in *C. albicans* have not been conducted, Sec62 and Sec63 are present in all eukaryotes and are essential to improving translocon affinity in yeast [64]. The ER-resident ATPase Kar2 facilitates the transit of nascent proteins into the ER lumen by enhancing translocation efficiency [72,73]. When *CaKAR2* was introduced into a *S. cerevisiae kar2* null mutant, the mutant was successfully complemented, indicating homology in gene function [72]. Many of these early secretory pathway genes, including *KAR2*, are essential for viability and involved in fundamental cellular processes and not specific virulence-related functions.

### 2.4. Protein Folding and Maturation

Secreted proteins in the ER undergo folding, maturation, glycosylation, and eventual targeting to the Golgi. Following translocation into the ER, the N-terminal signal peptide is cleaved by signal peptidase, a proteolytic enzyme [77]. In *C. albicans*, Spc3p, and Sec11p have been identified as subunits in the signal peptidase complex [54]. Spc3p is required for signal peptidase activity [54]. Using a *MET3*-repressible promoter, *C. albicans SPC3* has been demonstrated to be essential for viability [54]. Sec11 is a catalytic component of the signal peptidase complex and is the only catalytic subunit in *S. cerevisiae*, but its exact function has not been characterized in *C. albicans* [78]. 

Molecular chaperones and J proteins form the backbone of the protein folding process. Families of heat-shock proteins, particularly Hsp70, Hsp40, and Hsp90, function in the initial folding of the polypeptide [79,80]. Hsp70 has a conserved N-terminal domain with ATPase activity and a variable C-terminal domain reserved for peptide binding [63,81,82]. In yeast, the Hsp70-Hsp90 chaperone complex is responsible for protein folding in the cytoplasm, with Hsp70 folding the emerging polypeptide from the ribosome and Hsp90 continuing later folding [79,83]. Molecular chaperones are present throughout the secretory pathway and have recently been found to have a role in drug resistance, as *C. albicans* Msi3p is essential for cell growth and fluconazole resistance [72]. Chaperones in the Hsp40 family are referred to as J proteins due to the presence of a 70 amino acid J domain that contains the conserved histidine-proline-aspartate (HPD) tripeptide [27,84]. In *S. cerevisiae*, Ydj1, a member of the Hps40 family, regulates Hsp70 ATPase activity, influencing protein folding [84,85,86,87]. In addition, studies in *S. cerevisiae* have revealed several ER-resident J proteins, notably Sec63, Hlj1, Erj5, Scj1, and Jem1, but only Jem1p has been extensively investigated in *C. albicans* [27]. The Jem1p ortholog in *C. albicans* demonstrated a conserved function with Sc Jem1, interacting with Hsp70p in the ER lumen to aid in protein folding [27,88]. 

Yeast also displays an unfolded protein response (UPR) in response to an accumulation of unfolded or misfolded proteins in the ER lumen. In *C. albicans*, the UPR is controlled by inositol requiring enzyme Ire1p, a multi-signal transducer [56,89,90,91]. Under stress conditions, Ire1p initiates the production of the basic-leucine zipper (bZIP) transcriptional factor Hac1p to induce transcription of downstream UPR genes, facilitating both proper protein folding and protein translocation out of the ER into the cytoplasm for degradation [56,89,91]. Further details on the UPR have been reviewed in detail elsewhere [88]. 

In addition to the UPR, ER proteins are involved in maintaining a favorable environment for protein folding. A zinc finger transcription factor, Sfp1p, responds to carbon-conditional stress and oxidative stress in *C. albicans* [92,93]. Mutants lacking *SFP1* were found to be more resistant to oxidative radicals and macrophage killing [91]. Together with another transcription factor, Rtg3p, Sfp1p negatively regulates the transcription of stress response genes *CAP1*, *CTA4*, and *HOG1* [89,91,92,93,94]. Cap1p is a bZIP transcription factor that controls antioxidant gene expression and *HOG1* encodes a transcription factor that activates a MAP-kinase cascade to result in resistance to oxidative stresses [91,92]. Overaccumulation of reactive oxidative species (ROS) compromises cell wall integrity and affects the maturation of the proteins in the ER, impacting *C. albicans* virulence [95,96]. Furthermore, ER functioning requires tight regulation of ER lumenal calcium levels, which is maintained by Spf1p, a key P-type ATPase [97]. Deletion of *SPF1* disrupts downstream functions of *C. albicans* cell wall integrity, biofilm formation, and virulence due to the stimulated calcium influx [98]. *C. albicans spf1*Δ/Δ null mutants are hypersensitive to cell wall stressors such as Calcofluor white, and have decreased *SEC61* expression, interfering with the translocation of nascent proteins [96,98]. Secreted acid phosphatases derived from *C. albicans spf1*Δ/Δ null mutants exhibited glycosylation defects [96]. 

After secreted proteins are correctly folded, they are further modified through disulfide bond formation, glycosylphosphatidylinositol (GPI)-anchor addition, N-glycosylation, and O-glycosylation [17,27,28,89]. The process of disulfide bond formation is relatively unknown in *C. albicans*. In *S. cerevisiae*, the ER oxidoreductin 1 protein (Ero1) oxidizes an ER protein disulfide isomerase (PDI) and transfers a disulfide bond [99,100]. PDI, in turn, passes on the disulfide bond to substrate secreted proteins [97,98]. Accumulation of oxidized PDI leads to a negative feedback loop that results in the inactivation of Ero1 [98]. The addition of GPI-anchors to proteins in the ER results in embedding into the plasma membrane when secreted. Over 115 GPI-anchored proteins have been identified in *C. albicans*, although only 35% of them have known functions [101]. There has been limited research conducted on the mechanisms of GPI-anchoring in *C. albicans*, but the process is activated by a Ras-signaling pathway and involves a transamidase enzyme complex [102,103]. 

The glycosylation pathway is well-conserved in eukaryotes [104,105]. N-glycosylation refers to the attachment of glycans to a nitrogen atom on an asparagine residue, forming an N-glycated protein Glc3Man9GlcNAc2 in the ER [40,106]. The N-glycated protein is processed by α-glycosidases and mannosidases into an N-mannan protein, Man8GlcNAc2, which is targeted for the Golgi and elongated by proteins in the Bmt1 and Mnnproteinfamilies [104,106,107,108]. The Mnnproteins provide structural integrity to secreted proteins [108]. Studies of the *C. albicans* homologs of *S. cerevisiae CWH41* (α-glycosidase I), *ROT2* (α-glycosidase II catalytic subunit), and *MNS1* (α1,2-mannosidase) have highlighted ER glucosidase functions in cell growth and maintaining cell wall composition [40]. Similarly, O-mannosylation begins in the ER lumen with the attachment of a α-mannose to serine or threonine residues in a protein, and is necessary for GPI anchoring, and lipid, cytoskeletal and membrane stability [109]. O-mannosylation is catalyzed by protein O-mannosyltransferases (Pmt proteins), utilizing dolichol phosphate mannose in the process [110]. The *C. albicans* genome encodes five isoforms of Pmt proteins, Pmt1p, Pmt2p, Pmt4p, Pmt5p, and Pmt6p, which are closely related to cell wall components, as the *C. albicans* cell wall is enriched with glycosylated proteins [110,111]. *PMT2* is essential for viability, whereas mutants lacking *PMT1* have a mild defect in growth and substantial growth defects in the presence of hygromycin B, ketoconazole, and certain other stressors [108]. O-mannosylation must be strictly controlled as the misregulation of O-linked mannans can lead to increased sensitivity to cell wall active agents, defects in biofilm formation, and decreased virulence [111,112]. *C. albicans* mutants with defective mannosyl residues in *PMR1*, *MNN* and *MNN2* showed decreased virulence, demonstrating the effect of protein modification within the ER in pathogenesis [113,114,115,116]. Adhesins in *C. albicans*, such as the Als family of proteins, are also characterized by extensive post-translational modification through the classical secretory pathway. *C. albicans* Als1 – 7p and Als9p, which assist in adhesion to surfaces in various micro-environments in a highly complex manner, are characterized by an N-terminal signal sequence, GPI-anchor, and typically extensive N- and O-glycosylation [4,117].

## 3. ER-Golgi Secretion

### ER–Golgi Anterograde Transport

Modified and folded secretory proteins exit the ER for trafficking to the Golgi apparatus for further processing through a highly conserved anterograde transport pathway (Figure 1) [118,119]. This pathway involves coat protein complex II (COPII) vesicles that shuttle proteins from the ER to the Golgi and is well-studied in the model yeast *S. cerevisiae* but is considerably less understood in the fungal pathogen *C. albicans*. As demonstrated in *S. cerevisiae* and the budding yeast *Pichia pastoris*, mature proteins assemble at ribosome-free transitional ER sites (tER) on the ER, also known as ER exit sites (ERES), where budding of COPII vesicles occurs [120,121,122,123]. In *C. albicans*, the COPII protein complex consists of five main proteins: Sec23p, Sec24p, Sec13p, Sec31p, and the small Arf GTPase Sar1p [118,119,124,125,126,127]. In addition, Erv25p and Emp24p have also been identified as components of the COPII coat, although their functions are unknown [128]. 

The process of COPII coat recruitment has been studied in detail in *S. cerevisiae*. The guanine nucleotide exchange factor (GEF) Sec12p recruits and activates Sar1p by exchanging its bound GDP to GTP [129,130,131]. *C. albicans SAR1* is 78% identical to its *Sc SAR1* homolog [127]. The activated GTP-bound Sar1p exposes its N-terminal amphipathic α-helix for ER membrane attachment and facilitates the localization of the peripheral ER membrane protein Sec16 to the ERES [118,132,133,134,135]. Sec16 both stabilizes Sar1 in its GTP form and binds the multimeric Sec23/24 and Sec13/31 complexes, assembling the COPII coat [130,131,132,136]. Overexpression of Sc Sec16 resulted in an increased number of ERES and, consequently, increased protein secretion [128]. Homologs of *SEC16* and *SEC12* have been identified in *C. albicans* [137,138]. In *S. cerevisiae*, Sar1-GTP associates with the Sec23/24 complex to form an inner vesicle layer that selects and directly binds to cargo proteins, while the Sec13/Sec31 complex acts as an outer layer that adds structural integrity to the vesicle by interacting directly with the Sec23/24 complex [8,9,119,139,140]. Further research has suggested that Sec16, along with the membrane-associated Sar1-GTP, induces curvature in the ER membrane that allows for the formation and budding of the COPII vesicle [118,132]. Both Sc Sec16 and Sc Sec24 have been found to regulate COPII coat assembly and disassembly and prevent premature vesicle division [141].

Following its release from the ER membrane, the COPII vesicle tethers and fuses with the *cis*-Golgi, a process mediated by GTPases, transport protein particle (TRAPP) complexes, and soluble N-ethylmaleimide-sensitive factor activating protein receptor (SNARE) proteins. The Rab-type GTPase Ypt1p is essential to *C. albicans* vesicle docking and demonstrates 77% homology to *S. cerevisiae* Ypt1 [35]. Overexpression of a dominant-negative allele of Ypt1p results in *C. albicans* mutants with defects in the early secretory pathway, resulting in decreased secretion of virulence-associated aspartyl proteases and viability, supporting the role of Ypt1p in ER to Golgi trafficking [35,142].

In yeast, Ypt1 is regulated by the GEF activity of TRAPP tethering complexes, allowing Ypt1 to anchor to the *cis*-Golgi [143,144,145,146]. Four TRAPP complexes (I-IV) have been identified. TRAPPI and TRAPPIII are involved in ER to Golgi trafficking, TRAPPII plays a role in intra-Golgi transport and will be discussed later in the review, and TRAPPIV is proposed to assist in synthetic genetic interactions [143,147]. It was recently demonstrated that *S. cerevisiae* may possess only two TRAPP complexes in vivo, TRAPPII, and TRAPPIII, with only TRAPPIII participating in COPII vesicle tethering [141]. The appearance of TRAPPI in previous studies has potentially been attributed to experimental artifact [143,148]. TRAPPI and TRAPPIII both contain Trs20, Trs23, Trs31, Trs33, Bet3, and Bet5 subunits, but TRAPPIII comprises an additional Trs85 subunit [143,149]. *S. cerevisiae trs85* and *ypt1* null mutants display diminished cell growth and an accumulation of the marker protein GFP-Snc1 in the ER, suggesting that Trs85 and Ypt1 participate in preventing cell toxicity by allowing proteins to exit the ER [146]. Although specific studies have not been conducted, orthologs of genes encoding each TRAPPIII subunit have been discovered in *C. albicans* [150,151]. As described in *S. cerevisiae*, COPII docks at the *cis*-Golgi via an interaction between the Bet3 subunit of TRAPPI to the Sec23 component of the COPII coat [146]. SNARE complexes facilitate the uncoating and fusion of vesicles to the Golgi membrane, delivering cargo proteins into the *cis*-Golgi [119]. Sc Uso1p, together with Ypt1p, plays a role in the assembly of both vesicle-SNAREs (v-SNARE) and target-SNARE (t-SNARE) complexes [152,153]. In *C. albicans*, Bos1p, Sec22p, and Ykt6p have been identified as components of v-SNAREs involved in ER to Golgi transport [154,155,156]. *C. albicans ykt6*Δ/Δ mutants were unable to grow and form hyphae, indicating that *YKT6* is essential for viability [150]. In a large-scale screening campaign to identify anti-virulence compounds which inhibit *C. albicans* filamentation, Romo et al. [154], identified N-[3-(allyloxy)-phenyl]-4-methoxybenzamide. This compound down-regulated 702 genes associated with virulence and filamentation such as *SAP5*, *ECE1*, and *ALS3*, and up-regulated 618 genes, some of which are entirely uncharacterized. Out of the top 50 genes, *BOS1*, a putative v-SNARE of the ER and endosome-Golgi transport, was up-regulated, likely to promote vesicular transport and localization to the hyphal tip to compensate for the down-regulated effects suppressing filamentation [152]. 

## 4. Intra-Golgi Transport

Proteins destined for secretion are translated and glycosylated in the rough endoplasmic reticulum (RER) and packaged into COPII-vesicles for anterograde transport to the Golgi apparatus. The Golgi is the site of post-translational processing and modification, where protein glycosylation is completed. O-mannosylation in the Golgi plays a critical role in cell integrity and pathogenic host-fungal interactions. The adhesion and immunomodulation abilities of *C. albicans* are largely attributed to mannoproteins in its cell wall, which are modified through O-mannosylation [157]. Guanosine diphosphatase (GDPase) Gda1p and mannosyltransferases Mnt1p, Pmt1p, Pmt6p, and Pmr1p have been identified in the O-glycosylation pathway for *C. albicans* and are necessary for virulence [30,157,158,159]. The *C. albicans* genome also contains 10 virulence-associated secreted aspartyl protease genes (*SAP1* through *SAP10*) translated as preproenzymes, an inactive form of the mature enzyme containing a signal sequence to be cleaved and processed through the *trans*-Golgi resident protein Kex2p to produce the active enzyme [160,161]. 

The dynamic distribution of resident proteins within the Golgi cisternae and the highly conserved Golgi function between yeast and mammals demonstrate that proteins are processed from the *cis*-Golgi to the *trans*-Golgi via cisternal maturation [27,162]. In this model, secreted cargo proteins do not shuttle in vesicles but rather move through the Golgi as the *cis*-cisternae traverse through the Golgi stack, maturing into new *trans*-cisternae. As a *cis*-cisterna progresses through the stack, its population of resident proteins is replaced by *medial*- and eventually *trans*-Golgi resident proteins, suggesting that retrograde transport is required to recycle resident proteins from later to earlier cisternae after cisternal maturation. 

The mechanisms of intra-Golgi transport have been extensively studied in *S. cerevisiae* but are less well understood in *C. albicans*. Intra-Golgi retrograde trafficking of resident proteins from the *trans*- to *cis*-Golgi is mediated in part by COPI-vesicles and G-proteins in *C. albicans* [27,163]. Five Arf guanosine triphosphatase (GTPases), Arf1p, Arf2p, Arf3p, Arl1p, and Arl3p, have been identified to regulate protein trafficking and localize to the *trans*-Golgi [163,164,165]. Arf1p cycles between GTP-bound and GDP-bound states through regulation by guanine nucleotide exchange factors (GEFs) and GTPase-activating proteins (GAPs) and is required for COPI-coat assembly and COPI-vesicle budding in the Golgi membrane, a function conserved in eukaryotes [37,163]. Deletion of *C. albicans* Arl1p and Arf2p restrict invasive hyphae growth, demonstrating a dysregulation in protein localization [164,165]. In addition, *C. albicans* possesses several Rab GTPases (Ypt52p, Ypt53p, Vps21p, and a homolog of Ypt6p) and *trans*-Golgi localized vacuolar protein sorting complexes (Vps15p and Vps51p) that direct vacuolar and retrograde trafficking [165,166,167]. Although the specific role of Vps complexes has not been extensively studied in relation to intra-Golgi transport, Vps15p and Vps51p mutants exhibit abnormal retrograde trafficking and an inability to resist various stressors [167].

Tethering and fusion of COPI-vesicles to Golgi cisternae in yeast require TRAPPII, conserved oligomeric Golgi (COG) complexes and SNARE proteins. Previously demonstrated in *S. cerevisiae*, TRAPP II participates specifically in intra-Golgi and endosome-Golgi transport, containing a Trs120 subunit that recognizes and tethers COPI-subunits [168]. The assembly of TRAPP II in *C. albicans* is unclear, but is suggested to involve subunits Trs65p, Tca17p, and Trs130p due to the conservation of TRAPP II structure in yeast [169]. Cog2p and Cog6p mediate fusion of transport vesicles to the Golgi cisternae and have been briefly studied in *C. albicans* [27,55]. In yeast, the Cog Sec34/35p complex has been hypothesized to localize to the *cis*- and *medial*-Golgi and play a role in fusing the COPI-vesicle membrane to the Golgi membrane [170,171]. The SNARE fusion complex includes Sed5p and Sly1p, but their roles in *C. albicans* intra-Golgi secretion are unknown [172]. 

## 5. Pre-Vacuolar Secretion

Proteins that exit the trans-Golgi are either recycled back to the Golgi via retrograde trafficking pathways, traverse through the plasma membrane for exocytosis, or are transferred to the vacuole for further modifications, storage, or degradation. Originally it was believed that the destination was encoded in the glycosylation pattern on the protein, however convincing evidence has accumulated that structural features within the polypeptides themselves determine localization of the protein. The process that determines the pathway a protein takes appears to be saturable and thus is likely receptor-mediated [173]. These structural features in polypeptides allow interactions with or binding to receptors in vesicle membranes, which then move with the secretory vesicles, to be released either by changes in the milieu such as pH, or via processing by proteolysis. 

The pre-vacuolar compartment (PVC) functions as a nexus for vesicles arriving from the trans-Golgi and those derived from endocytosis destined to be stored or degraded in the vacuole. Vps21 is an endosomal protein involved in the trafficking of both vacuole-resident proteins and endocytosed proteins. It is a member of the Rab-GTPase family with sequence homology to mammalian Rab5 [174]. G.E. Palmer’s group cloned the *C. albicans* homolog and produced a null mutant *vps21*Δ/Δ. Loss of Vps21p-mediated trafficking results in mild reductions in stress resilience, hyphal growth and virulence [175]. However, in combination with loss of other endosomal trafficking-related proteins, including the endosomal Rab-GTPases Ypt72p or Ypt52p these defects are exaggerated [37,39]. A double mutant containing *vps21*Δ/Δ and a null mutation of *AP3*, which encodes a component of the coat complex involved in the direct trafficking of proteins from the Golgi to the vacuole, severely impairs the capacity of *C. albicans* to form hyphae, without major alterations in the morphology of the vacuole [175]. A triple mutant, *vps21*Δ/Δ *ypt52*Δ/Δ *aps*3Δ/- produces an aberrantly large vacuole (in the yeast form) and results in severe loss of vacuolar function [37]. This triple mutant had a more severe phenotype with defects in hyphal growth and morphology than double mutants. The relatively weak phenotype of individual mutants and the synthetic nature of these trafficking mutants suggest that there is redundancy in the trafficking pathways [176]. 

The ESCRT machinery is well conserved in all major eukaryotic organisms. Much of the early work done in *S. cerevisiae* has been confirmed in *C. albicans* and mammalian cells (reviewed in [177,178]). In fungi, the ESCRT complexes function to monitor environmental cues such as extracellular pH [179] and drive lifestyle changes such as switching from single cell to filamentous growth, by way of activating transcription factors, including Rim101. These transcriptional changes are important to *C. albicans* virulence [180]. However, it is also clear that some ESCRT functions that are required in virulence and pathogenesis are independent of Rim101p-regulated transcriptional changes [179].

Not surprisingly, the components of the ESCRT machinery have been frequently revealed in screens that were designed to find genes in the vacuole protein sorting (VPS) pathways. For example, Vps27p is a component of ESCRT-0. The *C. albicans* strain carrying a *vps27*Δ/Δ mutation grows normally and has a similar degree of filamentation as wild-type [181]. The null mutant has an iron sensitive phenotype and attenuated epithelial cell damage in in vitro assays. In an in vivo murine oropharyngeal candidiasis model, the null mutant has defects in initial oral colonization but not in maintenance of infection or immune evasion.

Screens for mis-localized vacuolar protein carboxypeptidase Y (CPY) in *S. cerevisiae* identified *VPS4* as a vacuolar protein-sorting gene, involved in the transport of proteins out of the pre-vacuolar compartment (Figure 1) [182]. Vps4 is an AAA-ATPase that is required for the sorting of endosomes to multivesicular bodies [181,183]. Vps4 was found to interact with yeast ESCRT [184]. The *C. albicans* homolog of *VPS4* was cloned based on predicted amino acid identity (>75%) and found it to functionally homologous to *S. cerevisiae VPS4* [21]. The *vps4*Δ/Δ null mutation in *C. albicans* results in accumulation of aberrant multilamellar vesicles that resembles those observed in *S. cerevisiae*, a hallmark of class E vesicle morphology [185]. Loss of Vps4p has minimal effect on the growth of *C. albicans* in rich media and has only modest growth retardation at high osmotic stress. Unexpectedly, the *C. albicans vps4*Δ/Δ mutant degraded more extracellular BSA than the wild-type or isogenic re-integrant strains. This is likely due to the missorting of the soluble serine protease carboxypeptidase Y, which is normally localized to the vacuole, to the extracellular space. Vacuolar proteases A and B (PrA and PrB, respectively) also appear to be missorted from the PVC to the extracellular space (S.A. Lee, unpublished data). Notably, however, there is disruption in trafficking of the secreted aspartyl proteases Sap2p and Sap4-6p, which in wild-type *C. albicans* are secreted into the extracellular space, but in the *vps4*Δ/Δnull mutant fail to be exported, implying a pre-vacuolar trafficking pathway [21,186]. A proteomic screen examined the soluble proteins found in the supernatants of planktonic cultures of *C. albicans* [187]. Comparing the *vps4*Δ/Δ mutant to the prototrophic control strain DAY185 revealed that the supernatant of the mutant strain had significantly reduced levels of several proteins that follow a canonical secretion pathway (ie. ER to Golgi to plasma membrane). However, proteins that do not follow a canonical secretory pathway, such as glycolytic enzymes and stress proteins, did not show significant reduction in extracellular concentrations.

The *C. albicans vps 4*Δ/Δ mutant also shows alterations in filamentation and biofilm formation. The mutant has shorter and more highly branched filaments that result in denser biofilms than the wild-type or re-integrant control strains. The density of the biofilm may account for the observed increased resistance to caspofungin [187]. Furthermore, in a mouse model of disseminated candidiasis, the *vps4*Δ/Δ mutant is attenuated in virulence and caused no renal pathology [186]. Comparing several other models of infection [188], the *vps4*Δ/Δ mutant is found to be defective in tissue damage of the oral epithelia and macrophage killing, but not in an in vitro uro-epithelial model of infection. Further, an in vivo murine model of vaginal candidiasis also shows no long-term differences in fungal burden or tissue damage. Taken together this suggests that *C. albicans VPS4* contributes to virulence in a tissue-dependent manner, reflecting its function in many sorting pathways [187]. 

*VAC1* is another gene identified in *S. cerevisiae* in a screen for mis-localized proteases. Like *VPS4*, mutations in *VAC1* result in secretion of CPY instead of proper trafficking to the vacuole [189]. Additionally, the *S. cerevisiae vac1*Δ/Δ strain has a partial defect in vacuole segregation during bud formation [190]. The *C. albicans vac1*Δ/Δ null mutant [191,192] shows defective endosomal vesicle transport, hypersensitivity to divalent cations, and defective hyphal growth. The vacuolar defect manifests itself as large vacuoles, enlarged hyphal compartments and sparsely branched hyphae [40]. It is avirulent in the mouse disseminated infection model [191].

*VPS1* is a pre-vacuolar secretory gene, also originally identified in *S. cerevisiae* where it encodes a GTPase that mediates the budding of clathrin-coated vesicles from the late Golgi. These vesicles are normally destined for the vacuole, but in the absence of Vps1, these vesicles bearing Golgi and vacuolar membrane proteins are not transported to the PVC and vacuole, but rather are misdirected to the plasma membrane [193,194]. The loss of function mutation of *C. albicans VPS1* results in lower proteolytic and lipolytic activity and a reduction in extracellular Sap2p, as well as a delayed filamentation and sparse biofilm formation, supporting a role in virulence [192].

## 6. The Role of the Vacuole in Secretion and Virulence

The fungal vacuole is a membrane-bound organelle with an acidic aqueous interior that, like the mammalian lysosome, contains hydrolases for the degradation of macromolecules. In addition, the fungal vacuole functions as the main storage compartment for cellular protein products including proteases, lipases, and other enzymes, as well as amino acids and cations. The acidic pH in the lumen of the vacuole is necessary for the processing of some precursor proteins, e.g., by triggering the autocatalytic maturation of pro-PrA (Proteinase A) that in turn is essential for the maturation of other hydrolases including pro-CPY (carboxypeptidase Y). Once fully processed these hydrolases are available for autophagy, a process in which building blocks of membrane and cytosolic proteins are recycled. The vacuolar pH may also induce the dissociation of ligands from their receptors during receptor-mediated endocytosis and vacuolar targeting. [173]. 

The low pH in the vacuole and other endocytic vesicles is maintained by a large, evolutionarily conserved [193], multi-subunit complex, the vacuolar membrane H^+^ATPase (V-ATPase) complex. The V-ATPase utilizes the energy of ATP hydrolysis to pump protons into the lumen producing a proton gradient, which is the primary driving force for the transport of most metabolites into the vacuole [172]. Mutations in the interacting subunits of this complex generally result in the complete loss of function of the V-ATPase; subsequent alkalization of the vacuole leads to a constellation of aberrant cellular functions that is the same regardless of which protein of the complex has been mutated. In yeast, this shared phenotype is called the *vma* phenotype. The hallmark of the *vma* phenotype is slow growth in neutral or alkaline media, sensitivity to calcium and the inability to grow on non-fermentable carbon sources [194].

The V-ATPase complex has two major components: V_0_ and V_1_. V_0_ is an integral membrane complex that forms the transmembrane channel through which protons are shuttled [195]. Disruption in *S. cerevisiae* of Vma3, one of the three polypeptides that form a hexameric ring, completely collapses vacuole acidification, prevents assembly of the remaining V_0_ complex, and produces significant defects in vacuolar biogenesis and protein transport [196]. *VMA3* has been examined in *C. albicans* using a conditional tetracycline-regulatable promoter system [194]. Repression of *VMA3* expression in *C. albicans* prevents the assembly of the V-ATPase complex leading to the alkalization of the vacuole. Secretion of aspartyl proteases and lipases, as well as filamentation was decreased under *VMA3*-repressed conditions. Further, virulence in an in vitro macrophage-killing assay was suppressed.

Vph1p is a part of the V_0_ complex that transfers protons from the cytosol to the hexameric ring [197,198]. *VPH1* is unique among the genes encoding of the V-ATPase complex in that it has an isoform, *STV1*, in both *S. cerevisiae* and *C. albicans*. The isoforms are localized to different subcellular compartments: Vph1p is found in the vacuolar membrane and Stv1p is found in endosomal membranes. In *S. cerevisiae*, complexes containing Stv1p are significantly less efficient at translocating protons than those containing Vph1p [199]. Because the endosomal *STV1* can partially compensate for *VPH1*, *C. albicans vph1*Δ/Δ strains do not display a full *vma* phenotype; instead, they grow near normally. However, the *vph1*Δ/Δ null mutant only weakly acidifies the vacuole [200] and shows an increased sensitivity to toxic metals [201]. As compared to wild-type, vacuolar physiology and morphology is irregular and secretion of Saps and lipases are significantly reduced. Filamentation is modestly altered, but virulence is substantially diminished [199]. On the other hand, loss of Stv1p does not result in major phenotypic changes when Vph1p is available. Thus, Vph1p is essential for full acidification of the vacuole and the Stv1p isoform cannot completely replace it.

V_1_ is the component of the V-ATPase complex found on the cytoplasmic side of the membrane. The core of V_1_ is a hexamer that has alternating catalytic or regulatory subunits. *VMA1* encodes the catalytic subunits where the hydrolysis of bound ATP occurs. Vma2p perform regulatory functions [194,202]. In *S. cerevisiae*, disruption of *VMA2* in results in strains that are defective in vacuolar acidification and H^+^ ATPase activity [203,204]. 

The role of Vma2p in stress response and virulence was investigated in *C. albicans* utilizing a tetracycline-regulated *VMA2* mutant [205]. Under repressive conditions mutant strains have a classic *vma* phenotype including pH-dependent growth rates and slowed growth in the presence of calcium and glycerol. The absence of Vma2p precludes the assembly of a functional V-ATPase. Repressed *VMA2* mutants display vacuolar abnormalities, both in acidification and morphology, and did not produce hyphae. The secretion of Saps and lipases was also partially inhibited. Vma2p is involved in sub-cellular trafficking during starvation-induced autophagy, as evidenced by disrupted cytoplasm-to-vacuole-targeting under repressive conditions. These deficits had substantial effects on the virulence of the *tetR-VMA2* mutant in a *C. elegans* model of infection, where hyphal-mediated killing was significantly reduced under repressive conditions.

Peripheral V_1_ proteins function to couple the catalytic V_1_ component and the transmembrane pore V_0_ component. *VMA5* in *S. cerevisiae*, encodes a peripheral subunit that is essential for the glucose-regulated assembly/disassembly of the V-ATPase [206,207,208]. In *C. albicans vma5*Δ/Δ null mutants are found to have vacuolar dysfunction, slowed growth and deficiencies in calcium homeostasis [209]. This mutant is hypersensitive to nitrogen starvation, fails to form filaments, and has weaker adhesion and biofilm formation; it is avirulent in the mouse model of systemic candidiasis. In *C. albicans,* a second peripheral protein, Vma7p, also bridges between V_1_ and V_0_. The deletion of *VMA7* (*vma7*Δ/Δ) leads to defective acidification of the vacuole, inhibition of growth in high pH media, disruptions in endosomal degradation and an increased sensitivity to toxic metal ions [210]. Secretion of Saps and lipases was not examined in these mutants. These two peripheral proteins of V_1_ provide unique, essential and non-redundant functions to the V-ATPase that have consequences for virulence.

The vacuole also has a role in the homeostasis of cytoplasmic cations and pH. The observation that V-ATPase mutants have not only alkalized vacuoles but also an acidified cytoplasm, suggests alkalized vacuoles signal endocytosis and degradation of plasma membrane proteins [211,212]. A major protein component of the *C. albicans* plasma membrane is plasma membrane H^+^ATPase 1 protein (Pma1p) [213]. This ATPase is normally localized in the plasma membrane and pumps protons out of the cytoplasm to maintain a neutral or slightly alkaline pH. Glucose activates the ATPase of Pma1p, resulting in a sharp increase in cytosolic pH, which precedes hyphal formation. Removal of Pma1p from the plasma membrane prevents the spike in pH that signals filamentation. Recent work by Ranes et al. [214] demonstrated that truncations of *C. albicans* Pma1p at the C-terminus leads to defects in proper localization and proton pumping activity of Pma1p. Loss of Pma1p function results in an acidification of the cytosol, and reduced filamentation. Interestingly, the loss of Pma1p function results in hyper-acidification of the vacuole without altering the secretion of Saps and lipases [214].

## 7. Post-Golgi Secretion

Post-Golgi vesicles use microtubule- or actin-built transport systems to reach specific sites at the plasma membrane where they fuse and release secretory proteins to the extracellular site [215,216]. In order to dock and fuse with the plasma membrane, the interaction between the vesicle-bound Rab GTPase Sec4p and the evolutionarily conserved exocyst complex is necessary [217]. *Ca SEC4* was isolated by Clement et al. and Mao et al. ([35,36], respectively). *SEC4* is essential in *C. albicans* and the loss of function mutant substantially accumulates post-Golgi vesicles [35,218]. Overexpression of the dominant negative *sec4 (S28N)* allele in *C. albicans* inhibited growth, aspartyl protease secretion and secretory vesicle release [35]. The secretory defect was more directly demonstrated in Lee et al. [139]. When the dominant negative *sec4* (S28N) allele of *C. albicans* was overexpressed, two plasma membrane transporters (Cdr1p and Ftr1p) accumulated intracellularly while the localization of the vacuolar membrane ABC transporter Mlt1p was unaffected [137]. 

Ca Bem3p is a putative GTPase-activating protein for Rho-type GTPase Cdc42p and has high similarity to *Sc* Bem3p. Sc Bem3p plays a major role in directing secretory traffic to sites of polarized growth by affecting endomembrane recruitment, polarized delivery of Sec4p as well as by controlling the size of the Bem3p-containing Spitzenkörper-like compartment. Similar to Sc Bem3p, overexpression of Ca Bem3p in *S. cerevisiae* was competent in clustering Sec4p [219]. These findings support the notion of a crosstalk between Cdc42 signaling and vesicle trafficking [218].

**Figure 2:**
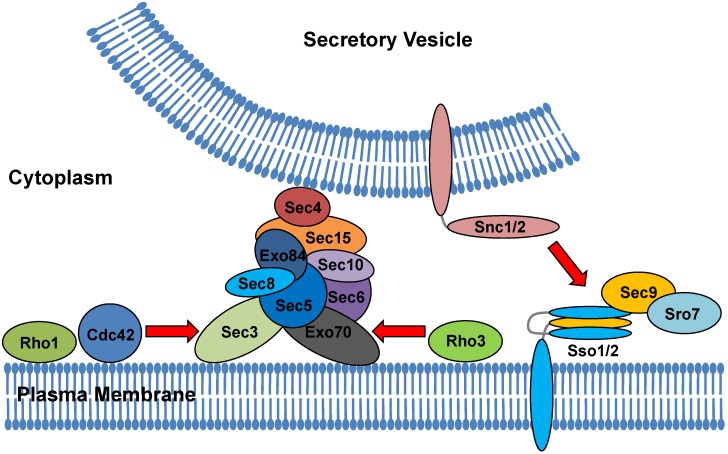
A model of the exocyst and SNARE complex. The exocyst is an octameric protein complex that marks the sites of exocytosis by facilitating the tethering and fission of late secretory vesicles to the plasma membrane. Based on data from *S. cerevisiae*, Sec3 and Exo70 proteins directly associate with phosphatidylinositol 4,5-bisphosphates in the plasma membrane. Assembly of the remaining exocyst components occurs as secretory vesicles arrive. The exocyst then functions to tether secretory vesicles to the exocytic sites and regulate SNARE assembly. The exocyst component Exo84 also interacts directly with Sro7 protein, which is a downstream effector of the small Rab-like GTPase Sec4, which regulates post-Golgi secretion. Additional regulation of the exocyst complex is performed by the Rho GTPases: Rho1, Rho3 and Cdc42. Snc1/2 proteins are the v-SNARES and Sso1/2 and Sec9 proteins are the t-SNARES required for final vesicle fusion to the plasma membrane just preceding exocytosis.

In *S. cerevisiae*, the exocyst is encoded by *SEC3, SEC5, SEC6, SEC8, SEC10, SEC15, EXO70,* and *EXO84* (Figure 2) [216]. This complex was first discovered by genetic and biochemical approaches in *S. cerevisiae*. Its postulated function is the tethering of post-Golgi secretory vesicles to the plasma membrane [216,217,220,221]. The model was developed based on discovered protein–protein and protein–phospholipid interactions [221,222,223,224,225,226,227] and *S. cerevisiae* mutation analyses [228,229,230]. A recently developed ectopic targeting assay in *S. cerevisiae*, in which eight exocyst subunits were expressed on the surface of mitochondria, revealed that most of the exocyst subunits were able to recruit other members of the complex. However, Sec4p and its guanine nucleotide exchange factor Sec2p are the main regulators of the assembly [231].

In *S. cerevisiae*, Sec3 is thought to be the only component of the exocyst that is able to provide a target for vesicle docking and fusion. This finding has been confirmed using the ectopic targeting assay [232]. Sec3 also binds to the t-SNARE protein Sso2 and promotes its interaction with another t-SNARE protein, Sec9 [229]. *Ca SEC3* encodes a protein 40% shorter than *Sc SEC3* with partial complementation of the *Sc sec3* mutant. *SEC3* is not essential but the null mutant has substantial growth and morphology defects similar to the temperature-conditional phenotype of *Sc sec3* mutants [23,228]. Sec3p is required for hyphal growth even if the first septin ring can be formed. Consistently, it interacts with the septins Cdc3p, Cdc10p, and Cdc11p. Sec3p localizes to a crescent on the surface of the hyphal tip [23,233].

In *S. cerevisiae,* proper localization of the exocyst depends on Sc Sec3/Exo70 association and their interaction with actin [223,230]. Recruitment of Sc Sec3 and Sc Exo70 is dependent on PI(4,5) P2 (phosphatidylinositol 4,5-bisphosphate) in the plasma membrane, and regulatory proteins Cdc42 and the Rho1 GTPase [222]. Vesicle fusion at the plasma membrane requires the SNARES Sec9, Snc1 or Snc2, and Sso1 or Sso2 [234]. Fusion is mediated by binding between specific pairs of cognate v-SNAREs and t-SNAREs on the vesicle and target membranes [235,236]. Rab GTP-binding proteins are required to facilitate formation of v-SNARE/t-SNARE complexes [235]. The t-SNAREs Sc Snc1/2 and the v-SNAREs Sc Sso1/2peach contribute to one helix, whereas the v-SNARE Sec9 contributes to two helices to the SNARE complex [236,237]. Tethering of the vesicle to the exocyst occurs first and is required for subsequent SNARE assembly, which allows fusion of the vesicle and target membranes leading to exocytosis [236]. The exocyst component Sc Exo84 also interacts directly with the SNARE regulator Sc Sro7, which binds to and activates the t-SNARE Sc Sec9 [238]. Exocytosis is strongly defective in *S. cerevisiaesro7* temperature-sensitive mutants. Sc Sro7 likely functions directly in late secretion by interacting with the activated (GTP-bound) form of Sc Sec4, thereby providing a functional link between vesicle arrival and assembly of the SNARE complex [237]. Supporting this notion, overexpression of the exocyst component Sc Sec15 and Sc Sro7 clustered Sec4 in internal compartments [239]. 

The functionally redundant Snc and Sso *S. cerevisiae* proteins arose through genome duplication. *C. albicans* contains only a single ortholog of each, as well as Sec9p [30]. A recent functional study in *C. albicans* indicated that *Ca SSO2* is an essential gene and required for secretion and cell division similar to *Sc SSO1/SSO2* [240]. When placed under a tetracycline promoter, the repressed *Ca tetR-Sso2* strain was defective in cytokinesis, with widened bud necks similar to the phenotype of *S. cerevisiae sso1*Δ and *sso2-1* temperature-sensitive mutant. This study also demonstrated functional homology of *C. albicans* and *S. cerevisiae SEC9*. Like in *S. cerevisiae*, *Ca SEC9* is an essential gene. In the absence of *Ca SEC9* gene expression, a conditionally repressed strain produced cells with wide bud necks and accumulated secretory vesicles, which was similar to the morphological defects found in the *Sc sec9-3* and *sec9-4* strains [228,241,242]. Analogous to the secretory defects described in these *S. cerevisiae* mutants, the *C. albicans* t-SNAREs Ca Sso2p and Ca Sec9p were required for secretion of the virulence-associated degradation enzymes such as secreted aspartyl proteases and lipases [240].

## 8. Extracellular Vesicles

Extracellular vesicles include exosomes that are formed within the endosomal network and are released upon fusion of multivesicular bodies with the plasma membrane [24,25]. They are small vesicles that are typically 30 to 100 nm in size [243,244,245]. Proteins enriched in extracellular vesicles and part of the *C. albicans* secretomeinclude glycan-binding proteins, membrane and cell wall proteins, cytosolic proteins with functions in pathogenesis such as adhesins or hydrolytic enzymes, cell organization, carbohydrate and lipid metabolism, response to stress (heat shock proteins; HSPs), and binding proteins for the ESCRT complex [22,243,246,247,248,249]. Lectin-binding motifs were conserved in all extracellular vesicles, although the proteins that bind to a given lectin were different [250,251,252,253,254]. Studies in *C. albicans* revealed that extracellular vesicles are part of a non-conventional protein secretory pathway that does not rely on signal peptides. Some of these have multiple functions and are also called moonlighting proteins, which are found in the extracellular environment and the cell wall [253]. The *C. albicans* GPI-anchored cell wall protein *ecm33*Δ/Δ null mutant has an altered cell wall and is avirulent. The proteomic analysis of proteins secreted by these cells identified a distinct pattern that was associated with an altered composition, size, and quantity ofextracellular vesicles. Specifically, the secretion of aspartic protease 2p (Sap2p) was compromised [255]. 

Fungal vesicles transport virulence-associated components to the extracellular compartment, suggesting that they are required for pathogenesis [243,246,248,256]. Few studies aimed at investigating *C. albicans* extracellular vesicle cargo proteins and their potential contribution to the pathogenesis of *C. albicans* infections have been conducted to date [257]. Proteins involved in *C. albicans* pathogenesis were detected in the extracellular vesicle cargoes. A recent study identified agglutinin-like protein 3 (Als3p), Sap8p and cell surface superoxide dismutase 6 (Sod6p) in vesicles of *C. albicans* strains cultivated in a nutrient-limited medium [256]. Recently, RNA molecules produced by *C. albicans* and other fungi have been found in extracellular vesicles [258].

*C. albicans’* extracellular vesicles contribute in biofilm matrix production and biofilm drug resistance [249]. Biofilms are a structure in which extracellular vesicles function not only in cell-cell communication but also in the sharing of nutrients. These vesicles are distinct from those produced by planktonic cells and have strong similarities in their composition to biofilm matrix material. The functions of biofilm extracellular vesicles were defined utilizing mutants defective in the ESCRT transport subunits. Most of these mutations cause reduced biofilm extracellular vesicle production. Biofilm matrix accumulation and drug hypersensitivity of these ESCRT mutants were reversed by addition of wild-type biofilm extracellular vesicles indicating that *C. albicans* biofilm extracellular vesicles have a pivotal role in matrix production and biofilm drug resistance [248]. 

An understanding of the mechanisms of vesicle release across the fungal cell wall is limited. Electron microscopy studies suggest an interaction with the cell wall [259]. The mechanisms of passage through the cell wall are largely unknown. Proteomic analysis of extracellular vesicles from *C. albicans*, and other yeast and fungi disclosed the presence of cell wall-degrading enzymes, suggesting that molecules present in the membrane aid in traversing the cell wall by hydrolysis of structural components [246,248,260,261,262]. 

Extracellular vesicles of *C. albicans* and other eukaryotic cells are thought to originate from the cytoplasm [254,263]. This notion is supported by evidence of cytoplasmic proteins lacking secretory signals in these vesicles [253,262]. Other studies indicate that multivesicular bodies and membrane budding may also participate in extracellular vesicle formation [259,264]. 

The ability of *C. albicans* vesicles to stimulate host cells is dependent on the lipid composition, since vesicles from phospholipid synthase mutants differentially stimulate cytokine production in macrophages [265]. Uptake in mammalian cells appears to depend on the type of target cell and one potential mechanism occurs through phagocytosis [262,263], if the recipient cell has phagocytic capabilities [266]. Macropinocytosis, clathrin-mediated endocytosis, and receptor–ligand interactions are alternative potential mechanisms through which extracellular vesicles transfer their content [267,268,269]. Membrane fusion of vesicles and mammalian plasma membranes require a similar fluidity, which was determined at an acidic pH [270,271] suggesting that this is a limiting condition for fusion [272]. 

## 9. Endocytosis and Endocytic Pathways

Endocytosis is needed for a cell to acquire nutrients, recognize extracellular signals and to regulate plasma membrane composition. This process begins with plasma membrane invagination, followed by the formation of endocytic vesicles that fuse with early endosomes. While most of the internalized content is sent back to the cell surface, the endosomal network sorts and facilitates transport of endosomes to cellular compartments, including the ER, Golgi and the lysosome for protein and lipid degradation [273]. 

**Figure 3:**
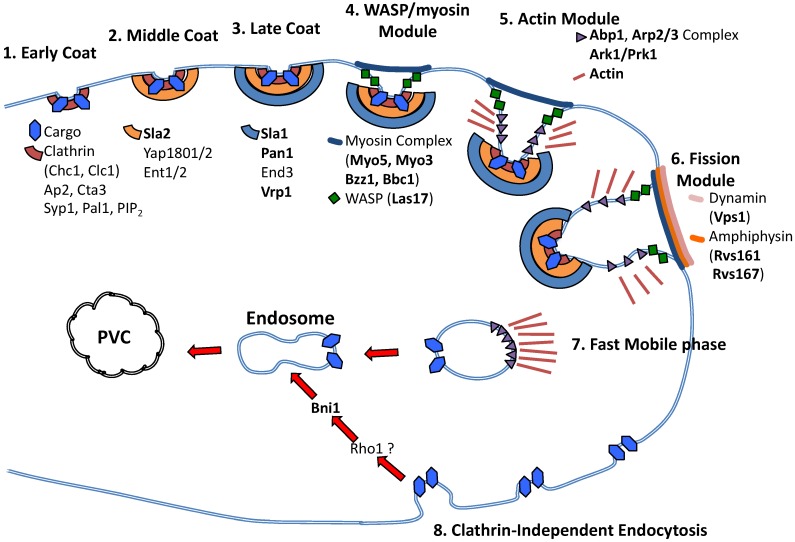
Clathrin-mediated and clathrin-independent endocytosis in yeast. Clathrin-mediated endocytosis is a highlyorchestrated, step-wise process that begins at the plasma membrane and leads to delivery of endocytic cargo through the endosomal pathway to the PVC (pre-vacuolar compartment). The clathrin-independent has fewer identified proteins but also leads through the endosomal pathway to the PVC. Proteins shown in bold have been studied in *C. albicans*, others are orthologous to ones studied in *S. cerevisiae*. Little is known about the role of clathrin-independent endocytosis in *C. albicans*.

In yeast, the most well-characterized endocytic process occurs via clathrin-mediated endocytosis (CME) (Figure 3), which has been studied in extensive detail. CME is mediated by >50 proteins in a temporallyordered manner (reviewed in Goode et al., 2015 [274]. The initial endocytic site is marked by cortical actin patches followed by clathrin coat assembly, actin assembly, membrane invagination, vesicle scission, and patch dissolution. The first early coat proteins to arrive in CME consist of clathrin, the AP-2p adaptor protein, Ede1, and Syp1, followed by Pal1. It is localized to the cell periphery developing bud necks in formation with other early coat proteins [275]. The early coat proteins Ede1 and Syp1 remain associated with the plasma membrane, while clathrin, AP-2, and Pal1 are internalized with the coat [276]. 

Maturation of endocytic vesicle formation occurs with recruitment of middle and late coat proteins. Sla2 plays a key role in the transition to middle coat endocytosis, which is followed by the epsins Ent1 and Ent2, which interact with the polarity-establishing GTPase Cdc42 [277]. Additional middle coat proteins include Yap1801 and Yap1802. Yap1801 and Yap1802 are clathrin-binding proteins, which bind to Pan1 and are involved in clathrin cage assembly in yeast [278]. Key late coat proteins include Pan1, Sla1, Prk1, End3, and Las17. Pan1 and End3 are recruited to endocytic sites prior to Las17 [279]. Las17 is recruited by Sla1 and drives actin assembly and forces membrane invagination [280]. End3 is an EF-hand Ca^2+^-binding protein that is required for the internalization of the endocytic vesicle and establishes proper actin patch and cell wall chitin localization [281]. The End3 C-terminus is the site of recruitment and binding by Pan1p to form a stable complex, enabling Pan1p phosphoregulation [282] Sc Pan1 is the only endocytic protein that is essential for viability, and contributes to actin cytoskeletal organization [283]. 

After formation of early and coat endocytosis modules which leads to polarized endocytic site localization and membrane invagination, there is an ordered and complex progression of mobile endocytosis via the WASP/Myo, amphiphysin, and actin modules, which have been well-described in *S. cerevisiae* [284]. 

Similar to *S. cerevisiae,* the majority of endocytosis in *C. albicans* is facilitated by clathrin-mediated endocytosis [285]. Several genes involved in endocytosis have been studied in *C. albicans* (Figure 3). *EDE1* is non-essential in *C. albicans*, and the null mutant grows normally and is filamentation-competent; endocytosis was not assayed [286]. Of the middle coat proteins, *Ca SLA2* contributes to filamentous growth [287]. Ca *sla2*Δ/Δ mutants are defective in fluid-phase endocytosis and polarized secretion, with mislocalization of lipid rafts normally trafficked to the hyphal tip [288]. Also, mislocalized are the lipid raft-associated protein Rvs167p and the cell wall remodeling enzyme β-1,3-glucan synthase Gsc1p. *Ca sla2*Δ/Δ mutants also appear to be defective in Rbt5p-mediated uptake of iron which relies upon endocytosis [289]. *Ca sla2*Δ/Δ mutants have reduced growth rates and enlarged, globular mother cells due to delays in cell cycle progression mediated by the morphogenesis checkpoint kinase Swe1p [290]. 

The key late coat gene *SLA1* has also been studied; *Ca sla1*Δ/Δ mutants produce shorter filaments in hyphal-inducing conditions and have a reduced number of cortical actin patches that fail to concentrate at the hyphal tip [291]. Interestingly, *Ca* *sla1/Δ* mutants displayed minimal defects in Lucifer yellow uptake but marked defects in FM4-64 internalization to the vacuole [292] and receptor-mediated endocytosis. Ca Sla1p activity is phospho-regulated by the cyclin-dependent kinase Cdc28p-Cln3p and the kinase Prk1p [293].

*Ca PAN1* has been studied to a limited degree, utilizing reverse genetic analysis of a *MET3* promoter-regulated conditional mutant, as *Ca PAN1* is an essential gene. The conditional *MET3p-PAN1/pan1* mutant, in repressing conditions, developed thick, swollen cells that did not filament normally and failed to endocytose FM4-64 [286]. The *C. albicans* homolog of *LAS17*, *Ca WAL1*, has also been investigated [286,293]. *Ca wal1*Δ/Δ mutants were notable for defects in endocytosis, vacuolar morphology, and hyphal development. While *Ca wal1*Δ/Δ mutants were able to initiate polarized morphogenesis, the majority of mutants formed pseudohyphae instead of hyphae.

More recently, it has been discovered that *S. cerevisiae* possesses clathrin-independent endocytic pathway(s), although details of these processes remain limited (Figure 3). The small GTPase Rho1p, the formin Bni1p, and the Bni1p co-factors and polarisome components Bud6p and Spa2p form the basis of a clathrin-independent endocytic (CIE) pathway, in an actin-dependent manner [294]. This pathway is further regulated by specific α-arrestin binding to components of the CIE pathway [295].

There is now indirect evidence for a Rho1p-mediated endocytic pathway in *C. albicans*, which is not dependent on Arp2/3, revealing unexpected differences from clathrin-independent endocytosis in *S. cerevisiae* [296,297]. Based upon a forward genetic screen for filamentation-defective mutants, a *Ca arp2*Δ/Δ mutant was found to be viable but unable to form hyphae; *Ca arp2*Δ/Δ and *arp2*Δ/Δ *arp3*Δ/Δ mutants were endocytosis-competent for Lucifer Yellow, and had only a modest delay of FM4-64 endocytosis to the vacuole [298]. In contrast, lack of *ARP2* in *S. cerevisiae* results in inviability or an extreme growth defect, and defective plasma membrane endocytosis. Detailed studies of the Ca Arp2/3 complex have revealed the existence of an Arp2/3-independent endocytosis pathway, which does not rely upon the major components of clathrin-mediated endocytosis but is actin-dependent [296]. The Ca Arc18p component of the Arp2/3 complex contributes to adhesion and biofilm formation; interestingly, the *arc18*Δ/Δ mutant had increased rates of Lucifer Yellow endocytosis and increased *RHO1* expression [296]. Little else is currently known regarding the role of endocytosis in virulence-related processes.

### The Endosomal Network

Endosomes play important roles in cell polarity, signaling, and lysosomal storage/degradation. Their functions are linked to the endosome’s maturation process. Maturation stages are defined by the presence of Rab GTPases coordinating membrane traffic and facilitating endosomal functions [299]. Unless destined for recycling back to the plasma membrane, endocytic vesicles traverse through the endosomal network, moving along actin cables to fuse with early endosomes [300]. These vesicles intersect with multiple anterograde secretory pathways depending on their processing path, which is defined by their cargo. They are in contact with the trans-Golgi network (TGN), which is mediated by the ESCRT/Vps4 (AAA-type ATPase) complex and are interacting with the ER [301]. This cargo-specific retrograde transport from the TGN, e.g., Vps10, depends on an intact retromer complex (reviewed in Schellmann and Pimpl [302]). After these processing steps, the late endosome is formed which then interacts with the pre-vacuolar compartment and finally the vacuole to facilitate degradation. Endosomal network maintenance is facilitated by the ER and the TGN. Both cell compartments promote the exchange of membrane components, provide enzymes, and assist with signaling.

In pioneering work done by Mellman and others, it has been demonstrated that endocytic trafficking requires a progressively acidified environment, from early to late endosomes and finally the vacuole (reviewed in [303]). Because of its effects on endosomal and vacuolar pH, *S. cerevisiae* V-ATPase contributes substantially to maintaining the pH-gradient needed for endocytosis [304]. *Sc* Nhx1p is a Na^+^/H^+^ and K^+^/H^+^ antiporter that localizes primarily to endocytic compartments, including the pre-vacuolar compartment (PVC) [305], and is required for trafficking of cargo out of the PVC to the vacuole [306]. Nhx1 transports Na^+^ into endocytic compartments in exchange for H^+^ and thereby mediates late endosomal/PVC lumenal pH [307]. In response to acidic pH, *S. cerevisiae nhx1* mutants have a reduced ability to alkalinize the cytosol and are defective in endocytosis. Thus, regulation of pH within endocytic compartments by V-ATPase, Nhx1p, and likely other proton pumps is required for efficient endocytic trafficking.

A wide variety of secretory mutants in *S. cerevisiae* display defects in endocytosis. The late secretory mutants *sec1*, *sec2*, and *sec5*, the Golgi-related secretory mutants *sec7* and to some extent *sec14*, and ER-related secretory mutants (*sec18* and *sec23*, but not *sec20* and *sec21*) were identified [308]. Mutants lacking *SEC18*, a SNARE chaperone involved in ER-Golgi transport, accumulated the endocytic marker Ste2p in small vesicles [309]. The pre-vacuolar secretory protein Vps1 also contributes to endocytosis; in a deletion mutant, internalization of vesicle-associated Snc1p and the lipophilic dye FM4-64 is impaired [310,311].

In *C. albicans,* a *pep12*Δ/Δ null mutant also showed clear defects in endocytosis. It is a predicted target membrane receptor (t-SNARE) involved in vacuolar transport, endocytosis, and secretion [312]. *Ca PEP12* is also required for biofilm formation and virulence in a mouse model of disseminated candidiasis. Genes from other pathways have also been implicated in endocytosis, e.g the plasma membrane exsosome-related protein encoded by *SUR7* [313], ADP-ribosylation factor GTPase activating protein Age3p [314], SH3-, and BAR domain-containing protein Rvs167p [291] and the membrane proteins Mon2p [315] and Ecm33p [316,317]. Finally, Ca Vps21p is a late endosomal Rab small monomeric GTPase involved in transport of endocytosed proteins to the vacuole as well as involved in filamentous growth and virulence in an in vivo mouse model of candidiasis [39,318]. 

## 10. Polarized Secretion in *Candida albicans* Hyphae

Polarized secretion is a key cellular process by which secretory vesicles are directed to specific sites in the plasma membrane to facilitate processes such as asymmetric formation of a yeast bud prior to mitosis, cytokinesis to produce a daughter cell, as well as formation of hyphae and hyphal growth [319,320]. *C. albicans* hyphae perform polarized growth from their tip [321]. This highly orchestrated process is enabled by multiple structures such as the Spitzenkörper, polarisome, and the secretory apparatus primarily found in the hyphal tip. Recent findings suggest that the organization of the secretory pathway facilitates optimized trafficking of secretory vesicle to the hyphal tip. In this model, the ER and Golgi move into the extending filament allowing reduced distances of travel within the tip region for secretory vesicle delivery [322]. Polarized secretion starts with a stream of vesicles accumulating in Spitzenkörper [323,324], an organelle of clustered vesicles that provides the material for hyphal growth [322]. Vesicles are then recruited by the polarisome and transported to the cell surface before they dock at the plasma membrane, facilitated by components of the exocyst complex and subsequent delivery of its cargo by SNARE-mediated membrane fusion [233]. While the exocyst complex and SNARE-mediated fusion are evolutionarily conserved in eukaryotic cells, their involvement in hyphal formation suggests potential pathogen-specific roles.

The growing tips of hyphae depend on rapid expansion of the cell membrane and wall, the organization of the cytoskeleton as well as trafficking of secretory vesicles [325]. Plasma membrane fusion with secretory vesicles ensures that (i) the vesicle membrane is incorporated into the plasma membrane, allowing expansion, (ii) the content of the vesicle is released into the extracellular space, and (iii) enzymes required for building the cell wall are delivered where needed [326].

The presence of mutants that develop buds and germ tubes efficiently but are unable to form true hyphae indicates that multiple systems of regulation are required for hyphal formation and growth. In *C. albicans*, septins form vital ring-like structures at the bud and pseudohyphal necks, but a much more diffuse array in emerging germ tubes of hyphae [327]. Without the septin genes *CDC3* and *CDC12*, *C. albicans* cannot proliferate [326]. Septins have been implicated in regulating polarized exocytosis. Deletion of the exocyst subunit gene *SEC3* is still permissive for normal germ tube formation, but the mutant is unable to maintain hyphal growth after assembly of the first septin ring, resulting in isotropic expansion of the tip. Deletion of either of the septin genes *CDC10* or *CDC11* caused Sec3p mislocalization [23]. Moreover, the septin Cdc3p binds to Sec3p and Sec5p, another exocyst component [23]. 

Another hyphal-specific gene is the cyclin *Hgc1*, which is needed to direct cyclin-dependent kinase (CDK) activity at the hyphal tip [328,329,330,331]. One role for the Hgc1p-CDK complex is to phosphorylate Sec2p, a guanyl-nucleotide exchange factor (GEF) for the small G-protein Sec4p. Sec4p is a vesicle-associated Rab GTPase that promotes polarized delivery of secretory vesicles [328], phosphorylation of the Exo84p subunit of the exocyst complex [328], and phosphorylation of septin proteins [332]. Moreover, Sec4p in its GTP-bound form mediates the tethering of vesicles with the exocyst component Sec15p [333]. Sec2p is also vesicle-associated but released to the cytosol upon vesicle tethering to the exocyst [334,335,336]. Thus, there are vesicle-associated and cytosolic pools of Sec2p. Both Sec2p and Sec4p are localized to the Spitzenkörper [227,328]. This localization of Sec2p is dependent on phosphorylation at S584 by Cdk1p [327]. In contrast to the localization of Sec2p and Sec4p, the exocyst components localize to a surface crescent [327].

The small GTPase Cdc42p is also required for hyphal growth. Mutants with reduced levels of this GTPase are unable to form hyphae and specific Cdc42p point mutants exhibit defects in the yeast to hyphal transition [337,338,339]. Expression of a constitutively active GTP-bound form of Cdc42p is lethal [337], and conditional expression of this activated mutant in hyphal cells resulted in cell swelling and reduced or altered polarity in *C. albicans* [340,341]. Cdc42p becomes stabilized at a defined site during symmetry breaking in an amplification step in the cell division process. When a new polarity site needs to be established in an already asymmetric cell, experimentally increasing the level of active Cdc42p on the plasma membrane results in mislocalization of the exocyst subunit Sec3p and an altered clustering of secretory vesicles. This new cluster of secretory vesicles is highly dynamic, until a new growth site can be established [342]. Cdc42p also promotes polarized recruitment of several polarisome components, including Spa2p and the formin Bni1p *in C. albicans*. Bni1p induces formation of actin filaments to guide the secretory vesicles to appropriate exosome sites in the plasma membrane [325]. Formation of the Spitzenkörper depends on the presence of the Bni1p, polarisome, actin filaments, and microtubules. [322]. In conjunction with these two organelles the Golgi is also found at the distal portion of the hyphae while in yeast cells it is located randomly throughout the cytoplasm [343,344]. Localization of the Golgi also depends on actin and Bni1p [343]. In the absence of Bin1p, the Golgi is scattered in small vesicles [343].

While Spitzenkörper and polarisome components are located in the apex of the hyphal tip, the sites of endocytosis occur at a slightly subapical zone [233]. This finding was suggested when clustering actin patches, a structure that occurs at an endocytic site was found in the subapical zone. Molecular modeling indicates that this orientation of sites of exocytosis and endocytosis helps to promote polarized hyphal tip growth [345].

In the final steps before membrane fusion, tethering of the vesicle to the exocyst occurs and is required for subsequent SNARE assembly. This complex then permits fusion with the plasma membrane and exocytosis. Previous studies have provided evidence for the role of SNAREs in hyphal development in filamentous fungi [346,347,348]. In *C. albicans*, the t-SNARE-encoding genes *SSO2* or *SEC9* demonstrated defects in hyphal growth and loss of Sso2p dissipates the Spitzenkörper, leading to an accumulation of secretory vesicles throughout the cell [240]. These findings support the notion that polarized secretion in hyphae depends on a properly functioning SNARE complex.

## 11. Non-Classical Secretion

Non-conventional secretion involves pathways in which proteins bypass the Golgi. In *C. albicans*, these pathways have not been extensively studied, though approximately one third of its extracellular proteins lack an N-terminal signal sequence, a hallmark of the conventional secretory pathway [33]. In contrast, approximately half of the *C. glabrata* secretome (grown in rich media in vitro) is comprised of proteins that lack N-terminal signal peptides [349]. In *C. albicans*, surface proteins lacking this peptide sequence have been found to associate with the fungal cell wall and include enolase, glyceraldehyde 3-phosphate dehydrogenase, 3-phosphoglycerate kinase, alcohol dehydrogenase, and catalase [69,350,351,352]. Further secretome analysis in yeast revealed that metabolic enzymes and heat shock proteins are secreted this way as well [353,354]. In extracellular vesicles isolated from *C. albicans*, cytoplasmic proteins including Hsp70p and enolase were detected [254].

In fungi, vesicle-mediated secretion is the prime route for non-conventional secretion [354]. Whether a protein is secreted via a single pathway and how the secreted vesicles pass through the fungal cell wall remains unknown [355,356]. A proposed mechanism suggests that extracellular vesicles originate from membrane reshaping and cytoplasmic subtraction [263]; however, multiple specific mechanisms may exist that have not yet been identified. Several extracellular vesicles have been described in fungi, including (i) exosomes, (ii) microvesicles, and (iii) Golgi-derived membrane vesicles [354]. Exosomes are secreted when a multivesicular body fuses with the cell membrane; microvesicles bud off the cell membrane; and Golgi-derived vesicles bud off the Golgi membrane and eventually fuse with the cell membrane, releasing their contents into the extracellular space [353]. The mechanisms for sorting proteins to these different vesicles are unknown, but environmental stimuli are thought to induce changes in the secretory proteins found in extracellular vesicles [67,354].

Non-secretory proteins are also able to enter the secretory pathway based on positive regulation by the N-terminal signal sequence and negative regulation by the conserved nascent polypeptide-associate complex (NAC) [28,356,357]. NAC associates with the nascent peptide-ribosome complex on the protein and prevents its integration into the ER; without it, proteins that lack an N-terminal signal sequence are still able to enter the conventional secretory pathway [358]. In *S. cerevisiae,* the NAC complex is composed of Egd1, Egd2, (orthologues of which are encoded by *C. albicans*) and Btt1 (not encoded by *C. albicans*) [240,359]. In *S. cerevisiae*, Btt1 is a paralog of Egd1 and expressed at a fraction of the level of Egd1, so selective modulation of NAC function may be a potential mechanism for non-signal sequence proteins to be secreted via the conventional pathway [28,360]. 

While the mechanisms of non-classical secretion in *C. albicans* have only been defined to a limited extent, it can be inferred that these pathways are important for pathogenesis based on the importance of their specific cargo proteins. Notable extracellular proteins that do not appear to be trafficked via conventional secretory pathways include metabolic enzymes such as enolase, glycolytic enzymes, chaperone proteins such as the Hsp70 family including Ssa2p, and elongation factors [187,254]. A number of these proteins are highly immunogenic and may contribute to pathogenesis [254,361]. Furthermore, biofilm-associated extracellular vesicles in *C. albicans* have been associated with delivery of extracellular matrix and contribution to antifungal drug resistance [249].

## 12. Conclusions and Future Directions

While the complex processes involved in secretion have been the subject of detailed study in the model yeast *S. cerevisiae*, there are important differences in these secretory pathways in *C. albicans,* which remains understudied. Although *C. albicans* and *S. cerevisiae* are genetically similar in terms of overall predicted genes, there are significant differences in their genomes, including size, differences in sexual cycle, ploidy, codon translation, and gene family expansions [362]. *C. albicans* is uniquely adapted to mammalian commensalism and has the ability to filament and cause infection. Evolutionarily, *S. cerevisiae* is much more closely related to *C. glabrata*, and distantly to *C. albicans,* which instead resides in the CTG clade. Notably, it has become evident that while many highly similar orthologs in these yeasts have divergent functions, conversely, some non-homologous genes have evolved convergent functions (reviewed in Whiteway et al. [363], with a remarkable degree of transcriptional rewiring evident [364]. Even minor differences in yeast secretion, which is overall highly conserved in eukaryotic cells, lead to distinctly different phenotypes (reviewed in Dellic et al. [27]). Thus, although secretion is highly conserved in eukaryotes, the transcriptional circuitry has been extensively rewired in *C. albicans.* Further work is needed to identify novel species- or clade-specific genes involved in the regulation of trafficking and secretion in *C. albicans*, which are necessary for polarized secretion, filamentation, and virulence of this opportunistic pathogen.
